# Developing evidence-based maternity care in Iran: a quality improvement study

**DOI:** 10.1186/1471-2393-8-20

**Published:** 2008-06-13

**Authors:** Siamak Aghlmand, Feizollah Akbari, Aboulfath Lameei, Kazem Mohammad, Rhonda Small, Mohammad Arab

**Affiliations:** 1 Department of Health Management and Economics, School of Public Health, Tehran University of Medical Sciences, Poursina Avenue, Tehran, 1417613191, Iran; 2 Mother & Child Health Research, Faculty of Health Sciences, La Trobe University, 324-328 Little Lonsdale Street, Melbourne, Victoria 3000, Australia; 3 School of Medicine, Urmia University of Medical Sciences, Jahad Avenue, Urmia 57147, Iran; 4 Department of Epidemiology and Biostatistics, School of Public Health, Tehran University of Medical Sciences, Poursina Avenue, Tehran, 1417613191, Iran

## Abstract

**Background:**

Current Iranian perinatal statistics indicate that maternity care continues to need improvement. In response, we implemented a multi-faceted intervention to improve the quality of maternity care at an Iranian Social Security Hospital. Using a before-and-after design our aim was to improve the uptake of selected evidence based practices and more closely attend to identified women's needs and preferences.

**Methods:**

The major steps of the study were to (1) identify women's needs, values and preferences via interviews, (2) select through a process of professional consensus the top evidence-based clinical recommendations requiring local implementation (3) redesign care based on the selected evidence-based recommendations and women's views, and (4) implement the new care model. We measured the impact of the new care model on maternal satisfaction and caesarean birth rates utilising maternal surveys and medical record audit before and after implementation of the new care model.

**Results:**

Twenty women's needs and requirements as well as ten evidence-based clinical recommendations were selected as a basis for improving care. Following the introduction of the new model of care, women's satisfaction levels improved significantly on 16 of 20 items (p < 0.0001) compared with baseline. Seventy-eight percent of studied women experienced care consistent with the new model and fewer women had a caesarean birth (30% compared with 42% previously).

**Conclusion:**

The introduction of a quality improvement care model improved compliance with evidence-based guidelines and was associated with an improvement in women's satisfaction levels and a reduction in rates of caesarean birth.

## Background

Around one million women give birth annually in Iran, with 90% receiving maternity care in hospital. Maternal mortality is still high compared with rates in developed countries (37.5 per 100000 live births), as is neonatal mortality (16.9 per 1000 live births), and the caesarean birth rate is close to 40% [1]. Of the 295 registered maternal deaths in 2005, 87.6% occurred in hospitals and 60% were found to be related to medical errors [2]. Despite many advances in the Iranian health care system over recent decades, these statistics alone show that there is still much room for improvement in the quality of maternity care [3].

Worldwide, several methods have been used for improving the quality of maternity care in hospitals, such as clinical practice guidelines (CPGs), clinical pathways, and clinical audit [4-11]. Almost all of these methods have their origin in evidence-based practice (EBP) to apply the best evidence in clinical care [12]. EBP is simply the integration of the best available research-based evidence, clinical expertise, and patient needs, values and preferences to develop a system of quality care [13]. Quality improvements thus require professional consensus about implementation of research-based clinical evidence, and attention to patient needs, values and preferences [14].

Although EB practice has been recognised as necessary for quality care in developed countries, it has often been conceptualised in terms of research-based clinical evidence and less attention has been paid to patient needs and preferences [15]. Yet service quality has been defined as meeting or exceeding service users' needs [16,17]. Patient satisfaction has indeed been recognised as an important outcome measure for the quality of health care since the late 1980s [18]. Furthermore identifying patient needs and requirements has been judged essential for both measuring and improving quality of care [19].

In order to improve the quality of maternity care at an Iranian Social Security Hospital serving a poor population (Fayazbakhsh Hospital), we measured the impact on women's satisfaction and caesarean birth rates of a multifaceted intervention to improve uptake of selected EB practices, utilising a before-and-after study design.

## Methods

Ethics approval to conduct the study was granted from the Ethics Committee of Tehran University of Medical Sciences and Urmia University of Medical Sciences (dated 20 September 2005) after submitting the information that would be given to the participants. All participants gave their written consent prior to the interviews and before-and-after surveys.

A four-step process was utilised to develop, implement and evaluate more evidence based maternity care at the study hospital [20].

### Step 1: Selecting evidence-based practices

In October 2005, a small team of health care providers (including an obstetrician, a neonatologist, an anaesthetist, and four midwives) was formed at the maternity ward to oversee the improvement. This team identified the most important EB practices from the following sources:

#### Women's needs, values and preferences

Between 31 January and 4 February 2006, the midwives of the team conducted in-depth structured interviews with women following birth (n = 18) to identify their needs and requirements using 11 open-ended questions (Table 1). The conduct of the interview had been standardised with the use of a detailed flowchart to be confident about consistency and reliability [see Additional file 1].

**Table 1:** The main open-ended questions of the interview with women in the postpartum unit, Fayazbakhsh hospital. The questions are designed based on 5Wh1H format (who, what, when, where, why, and how questions)

**No**	**Question**
1	Are you satisfied with type of your birth you have just had and why*?
2	Which type of birth would you prefer for next time and why?
3	Why did you choose this hospital?
4	Why did you not choose another hospital closer to your home?
5	Which experiences were positive during the hospital stay and why?
6	Which experiences were negative during the hospital stay and why?
7	Are there other services that you expected to receive why and how?
8	Who do you remember and why?
9	Which places do you remember and why?
10	Which moments do you remember and why?
11	Do you have any recommendation for better services in future?

*Patient needs and requirements are only identified by 'Why' questions

Through these interviews, fifty-four needs and requirements were identified and numbered. Subsequently, the team members helped a subgroup of the interviewees to rank identified needs and requirements using analytical hierarchy process (AHP) [21]. Pairwise comparison of identified needs and requirements using a 1–9 scale was the basis of AHP, with the 20 highest-ranked needs and requirements (comprising 70% cumulative weight) finally selected for further attention (Table 2).

**Table 2:** The highly-ranked women's needs and requirements weighed by analytical hierarchy process (AHP)

**Needs and requirements**	**% Relative weight**	**% Cumulative weight**
1. Well-being of baby	9.0	9.0
2. Well-being of women	6.4	15.4
3. Low-pain labour	5.6	21.0
4. Caring and sensitive staff	4.3	25.3
5. Frequent monitoring	4.2	29.5
6. Privacy during birth and vaginal examination	3.8	33.3
7. Quick response to requests	3.1	36.4
8. Labour and childbirth education	2.9	39.3
9. Provision of comfort	2.9	42.2
10. Listening to the fetal heartbeat	2.8	45.0
11. Vaginal birth	2.8	47.8
12. Companionship after birth	2.7	50.5
13. Immediate opportunity to see the newborn	2.5	53.0
14. Bed linen changed frequently	2.5	55.5
15. Improved hospital facilities	2.4	57.9
16. Painless vaginal examination	2.3	60.2
17. Short labour	2.2	62.4
18. Helping mother with breastfeeding	2.2	64.6
19. Clean labour ward	2.2	66.8
20. Quick admission	2.0	68.8

* Cumulative weight demonstrates that 37% of the most important needs and requirements (20 of 54) address about 70% of women values and preferences.

#### Research-based clinical evidence

The team reviewed the most well-known EB clinical practice guidelines: NGC (National Guideline Clearinghouse) and NICE (National Institute for Clinical Excellence) [22,23]. The AGREE (Appraisal of Guidelines Research & Evaluation) Instrument was then used to assess the quality (internal and external validity) of the guidelines. AGREE consists of six domains with each domain intended to capture a separate dimension of guideline quality such as scope, clarity, and applicability [24]. Twenty-eight high-quality clinical recommendations were selected, nine of which were already implemented routinely, but 19 of which had not been followed at the study hospital (Table 3).

**Table 3:** Evidence-based clinical recommendations.

	**Not implemented routinely prior to intervention (n = 19)**	
	
**Implemented routinely prior to intervention (n = 9)**	**Selected for intervention (n = 10)**	**Not selected for intervention (n = 9)**
Amniotomy unless contraindicated	Admission in labour phase	Elective episiotomy
Nurse auscultory monitoring	Adequate pain relief (only by parenteral analgesics)	Vaginal birth after caesarean birth
Continuous electronic fetal monitoring-external (EFM-ext), if indicated	Non-use of routine enema	Restriction of elective caesarean birth
Documentation of progress of labour	Companionship (only after birth)	Alternative position for delivery
Regular cervical exam	Mobility during the first stage of labour	Continuous electronic fetal monitoring-internal (EFM-int), if indicated
Chart evaluation	Oral fluids	Amnioinfusion for meconium treatment and/or oligohydramnios
Operative vaginal delivery, if indicated	Remedial techniques in uteroplacental insufficiency or cord compromise	Vibroacoustic test or scalp stimulation
Prevention of postpartum haemorrhage	Management of arrest disorders	The scalp pH test
Management of high-risk situations such as preterm and post term labour, bleeding, gestational diabetes and hypertension	Management of protraction disorders	Fetal Pulse Oximetry (FPO)
	Active management of the third stage of labour	

The left column shows the evidence-based clinical recommendations implemented routinely at the hospital. The next two columns display the result of professional consensus about which practices would be adopted in the new care model and which would not be adopted in the new model

#### Professional consensus

Using a Delphi technique, the physicians of the team tailored the 19 selected EB clinical recommendations to the conditions of the maternity ward according to four criteria (availability of resources, the physical environment of the maternity ward, clinical experience and culture, and correspondence with women's needs and requirements) [13]. To this end, the 19 recommendations were separately ranked for each criterion using a 1–5 scale by the physicians. Each ranking was continually modified until all the physicians achieved a consensus about the order of the recommendations. Finally, 10 of the 19 EB recommendations not already routinely implemented were chosen as the basis for improving maternity care (Table 3).

### Step 2: Assessing current care

We assessed women's satisfaction, clinical adherence to EBP, and the rate of caesarean birth at the hospital prior to implementation of the new care model.

The team conducted a baseline survey with a representative sample of women, who had given birth at the study hospital within the previous year, to appraise their level of satisfaction with given services. To this end, a self-completed questionnaire, using a Likert scale (1 for 'poor' to 5 for 'excellent'), was designed based on the previously identified 20 needs and requirements [21]. The questionnaire was piloted with 15 women. From piloting, the variance related to women's preferences was estimated (= 1.92) and the required sample size was calculated (n = 82 with α = 0.05, S^2 ^= 1.92, and d = 0.3). After modifying three questions, the final questionnaire provided scale scores ranging from 0–21 and was found to be reliable, with a Cronbach's α of 0.90 indicating very high internal consistency [25].

A random sample of 100 women was drawn up from the list of all women who had given birth in the previous 12 months (= 2213) using a table of random numbers. Given low levels of education and other cultural factors, the team decided to visit women in their homes in order to explain the study and support women to complete the questionnaires. A detailed flowchart of the process for visiting women was used to increase the reliability of the survey [see Additional file 2].

Between 23 July and 19 September 2006, the questionnaires were voluntarily completed by participants (= 89) at home (response rate = 89%). Two women had moved, one woman declined to take part and eight women were unable to complete the questionnaire due to literacy problems. Analysis of the survey was undertaken calculating the mean and variance of the responses to each question.

The team members also assessed clinical adherence to EBP in current care by reviewing the inpatient records of surveyed women and identifying practices that were inconsistent with the 10 selected EB recommendations. Mode of birth among participants was also noted in order to identify the proportion who had experienced a caesarean birth.

### Step 3: Designing the new care

**Figure 1:**
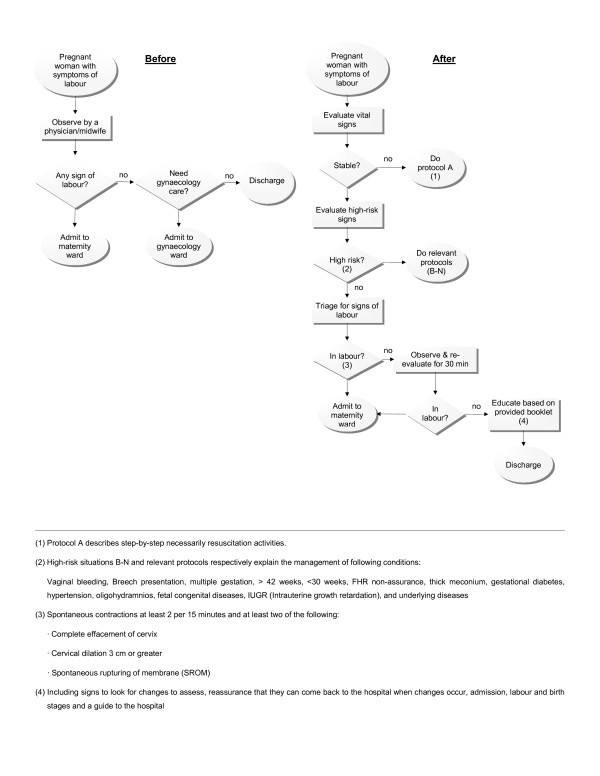
**The admission process of maternity care in Fayazbakhsh hospital in before (left) and after (right) introduction of the new model of care**: In the new model an evidence-based clinical recommendation namely, admission in labour phase, as well as a function including, labour education, have been introduced into the process.

As only five women's identified needs and requirements were supported by EB clinical recommendations (well-being of woman and baby, vaginal birth, companionship, and low-pain labour), the team radically redesigned maternity care also based on the remaining 15 identified needs and requirements along with the 10 EB clinical recommendations by developing a process flowchart of maternity care. An illustration of just one aspect of the flowchart for the new care model is shown in Figure 1. This demonstrates how the admission process was redesigned based on the selected EB recommendations as well as women's needs and requirements.

Physicians – 20 obstetricians, 6 anaesthetists and 4 neonatologists (90% of total physicians) – then reviewed the ideal process step-by-step and planned for realistic contingencies [26]. Over a one-month period, the physicians and midwives then participated in training courses and workshops which included EBP and quality improvement methods [27].

### Step 4: Implementing and evaluating the new care

Between 20 February and 16 March 2007, satisfaction data were again collected from a random sample of 100 women following the implementation of the new care model. For a period of 25 days, four women were randomly selected from all women who had given birth each day using random numbers generated by a relevant website, and they completed the same questionnaire with the same protocol previously used at baseline. Six initially selected participants who were unable to complete the questionnaire due to literacy problems were each replaced by further random selection form the day's births. One hundred fully completed questionnaires were thus obtained. Differences in women's satisfaction before and after the intervention were assessed using the Mann-Whitney non-parametric test in SPSS 13.0 [28].

The team members again reviewed the inpatient records of participants to identify the level of clinician adherence with the new maternity care model as well as their clinical outcomes (vaginal or caesarean birth). We calculated the relative risk (RR) and 95% confidence interval for caesarean birth in those women for whom the new maternity care model was not followed.

## Results

### Women's satisfaction

Prior to implementation of the new care model, women's satisfaction with care indicated low to moderate levels of satisfaction (mean < 3.6), on 15 of the 20 items. Statistical comparison of women's satisfaction levels before and after the intervention showed a significant increase in satisfaction for sixteen of the twenty needs and requirements. Just four items showed no significant improvement in women's rating – painless vaginal examination, low-pain labour, short labour and clean maternity ward – all of which had exhibited high levels of satisfaction even before introduction of the new model (Table 4).

**Table 4:** Women's satisfaction levels assessed before and after introduction of the new model of maternity care

		**Satisfaction level**				
			
		**Before**		**After**		
			
**No**	**Needs and requirements**	**Mean**	**Variance**	**Mean**	**Variance**	**P-value (two dimensional) with Mann-Whitney test**
1	Provision of comfort	3.5	0.77	4.57	0.29	< 0.0001
2	Well-being of woman	3.50	0.66	4.63	0.28	< 0.0001
3	Painless vaginal examination	4.41	0.45	4.29	0.63	0.371
4	Vaginal birth	2.47	0.50	4.41	1.31	< 0.0001
5	Companionship after birth	2.64	0.64	3.50	2.61	< 0.0001
6	Listening to the fetal heartbeat	3.46	0.66	4.85	0.17	< 0.0001
7	Immediate opportunity to see the newborn	3.54	0.61	4.79	0.34	< 0.0001
8	Low-pain labour	4.37	0.59	4.22	0.49	0.063
9	Quick response to requests	3.46	0.68	4.50	0.30	< 0.0001
10	Helping mother with breastfeeding	3.41	0.65	4.40	0.76	< 0.0001
11	Caring and sensitive staff	3.40	0.61	4.54	0.32	< 0.0001
12	Labour and childbirth education	4.30	0.62	4.91	0.10	< 0.0001
13	Well-being of baby	3.30	0.65	4.73	0.24	< 0.0001
14	Bed linen changed frequently	3.50	0.66	4.67	0.22	< 0.0001
15	Privacy during birth & vaginal examination	3.46	0.63	4.33	1.15	< 0.0001
16	Clean maternity ward	4.22	0.74	4.54	0.29	0.032
17	Improved hospital facilities	2.78	0.82	3.98	1.64	< 0.0001
18	Quick admission	3.40	0.87	4.68	0.37	< 0.0001
19	Short labour	4.29	0.80	4.43	0.45	0.536
20	Frequent monitoring	3.58	0.56	4.57	0.33	< 0.0001

### Clinician adherence to evidence-based care

None of the selected EB practices had been routinely followed before the intervention. After the new care model was introduced, clinicians complied with the new EB guidelines in the care of 78 participants (78%). The EB practice that had most often been missed was the new protocol for admission in labour (68%). In 67% of these instances of non-compliance, women experienced a caesarean birth.

### Caesarean birth

Prior to implementation of the new model of care, 42% of participants had a caesarean birth. Following its implementation 30% of women had a caesarean birth. In the 22 women for whom the new care model was not followed, the relative risk of caesarean birth was significantly higher (RR = 3.55, 95% CI: 2.07–6.07) (Table 5).

**Table 5:** The clinical outcome of clinical adherence to the new care model*

**Clinical Outcome**	CB**	VB***	**Total**	**% of CB**
**Non-adherence to the new maternity care model**	15	7	22	68.2
**Adherence to the new maternity care model**	15	63	78	19.2

**Total**	30	70	100	30

*The relative risk (RR) of caesarean birth is 3.55 (95% CI: 2.07–6.07) among women of clinicians not adhering to the new care model

** Caesarean birth

*** Vaginal birth

## Discussion

Implementation of the new maternity care model improved clinician compliance with evidence-based guidelines and was associated with an improvement in women's satisfaction levels and a reduction in rates of caesarean birth. Whilst promising, these findings need to be interpreted cautiously primarily because of limitations for attributing causality in the before-and-after design used here [29]. Thus, although our findings are consistent with those of other recently published studies [15,30,31], the impact of the intervention would be more appropriately studied in a well-designed randomised trial. Whether the method and findings of the study have value in other settings and with other clinical processes will only be seen in future studies.

Most of the quality improvement literature emphasises the involvement of people who know the process best (the process owners) as essential for eliciting the best information and for increasing the participation and commitment of quality improvement team members [32]. However there are some limitations. A dual role as caregiver and quality improvement team member can be a source of bias. In Step 1, the purpose of our interviews with women was to develop understanding of patient needs and requirements (not to assess their satisfaction with care). At this stage caregiver involvement was unlikely to introduce bias. However, the involvement of the midwives during the collection of satisfaction data (in Steps 2 and 4) may have been a source of bias in the study. We tried to increase the internal validity of the study through midwife training, assurances to women about the purpose of the study, standardisation of the survey process and supervision of the survey and data analysis by the authors who were external to the quality improvement processes [29].

The identified women's needs and requirements had a major role in design of the new care model. Available EB guidelines alone do not guarantee patient satisfaction or high quality of care. In this study women identified that they did not want support during labour and birth from their partner or a family member, but rather from their professional caregivers. Despite research evidence that supports the role for companionship during birth [33], women's stated preference was for non-professional support from their partners or family members, immediately after the birth. Thus the new model adopted a modified evidence-based guideline appropriate to the expressed wishes of the women attending the study hospital. In addition, professional consensus about areas of practice on which to focus improvement efforts largely ensured adherence to and smooth implementation of the new model of care. Our experience suggests that care designers should always consider *all *the relevant sources of evidence, including patient needs and requirements and professional consensus, in any EB initiative [34].

It appears that participation and raised awareness among clinicians played an important role in successful implementation of the new care guidelines. However, in around one in five cases compliance with the new model was not achieved. Changing care practices is always a time-consuming and ongoing process requiring much organisational support [35]. Lack of time and experience, conflicts between professional groups and/or generations, cultural and psychosocial characteristics of the community, the physical and technical environment, rights and rules, and fear of poor outcomes may all be barriers to the adoption of EB practices [36].

In our study it was of interest that caesarean birth was more likely to be associated with non-compliance with admission guidelines, that is, women being admitted who were not in active labour, did not have effective contractions and did not respond well to stimulation – a set of circumstances that may predispose to a decision to perform a caesarean. Alternatively, for women who attended hospital late in labour there may simply have been less opportunity for medical intervention.

## Conclusion

This multi-faceted intervention to translate appropriate evidence into practice appears to have had an important and positive impact on maternity care provision in the study hospital. Our study has demonstrated both that women are well aware of their needs and that identifying patient needs and requirements can play a major role in designing and measuring quality improvements. Equally, attention to professional consensus about priorities for EB practices appears to improve implementation of new care models and reduce clinician resistance to change. This study may also provide food for thought for health care policy-makers and care providers who are looking for better strategies to bring about evidence-based and patient-centred care.

## Competing interests

The authors declare that they have no competing interests.

## Authors' contributions

SA developed study design, administered the study team at the study hospital, performed data management and interpretation, and drafted the manuscript for publication. FA, AL, KM, RS and MA provided scientific advice on the design and implementation of the study, analysis and interpretation of the data as well as in drafting the manuscript. All authors read and approved the manuscript.

## Pre-publication history

The pre-publication history for this paper can be accessed here:



## References

[B1] Ministry of Health and Medical Education (MHME) of Iran (2004). The Report of the Health Indicators in 2003.

[B2] Motlagh MI (2006). Iran on the Mirror of Statistics.

[B3] Tita ATN, Stringer JSA, Goldenberg RL, Rouse DJ (2007). Two decades of the safe motherhood initiative: Time for another Wooden Spoon award?. Obstet Gynecol.

[B4] King JF (2005). A short history of evidence-based obstetric care. Best Pract Res Clin Obstet Gynaecol.

[B5] Smith H, Brown H, Hofmeyr GJ, Garner P (2004). Evidence-based obstetric care in South Africa: influencing practice through the 'Better Births Initiative'. S Afr Med J.

[B6] Ransom SB, McNeeley SG, Yono A, Ettlie J, Dombrowski MP (1998). The development and implementation of normal vaginal delivery clinical pathways in a large multihospital health system. Am J Manag Care.

[B7] Ransom SB, Studdert DM, Dombrowski MP, Mello MM, Brennan TA (2003). Reduced medicolegal risk by compliance with obstetric clinical pathways: A case-control study. Obstet Gynecol.

[B8] Fox S (2004). All-Wales clinical pathway for normal labour: a way to reducing unnecessary intervention. RCM Midwives.

[B9] Graham W, Wagaarachchi P, Penney G, McCaw-Binns A, Antwi KY, Hall MH (2000). Criteria for clinical audit of the quality of hospital-based obstetric care in developing countries. Bull World Health Organ.

[B10] Mercer SW, Sevar K, Sadutshan TD (2006). Using clinical audit to improve the quality of obstetric care at the Tibetan Delek hospital in North India: a longitudinal study. Reprod Health.

[B11] Ronsmans C (2001). What is the evidence for the role of audits to improve the quality of obstetric care?. Stud HSO P.

[B12] Stevens KR (2001). An introduction to evidence-based practice. Newborn Infant Nurs Rev.

[B13] Porter-O'Grady T, Malloch K, Porter-O'Grady T (2005). A new age for practice: creating the framework for evidence. Introduction to Evidence-based Practice in Nursing and Health Care.

[B14] Sanares DC, Heliker D, Malloch K, Porter-O'Grady T (2005). A framework for nursing clinical inquiry: pathway toward evidence-based nursing practice. Introduction to Evidence-based Practice in Nursing and Health Care.

[B15] Chaillet N, Dumont A (2007). Evidence-based strategies for reducing cesarean section rates: a meta-analysis. Birth.

[B16] Iacobucci D, Ostrom A, Grayson K (1995). Distinguishing service quality and customer satisfaction. J Cons Psychol.

[B17] Morand P (1995). Partnering with customers by capturing their voice. J Electron Manuf.

[B18] Williams B (1994). Patient satisfaction: a valid concept?. Soc Sci Med.

[B19] Aharony L, Strasser S (1993). Patient satisfaction: what we know about and what we still need to explore. Med Care Rev.

[B20] Sakala C, Corry MP (2001). What is evidence-based health care?. J Midwifery Women Health.

[B21] Chaplin E, Terninko J (2000). Customer driven healthcare: QFD for process improvement and cost reduction.

[B22] Institute for Clinical Systems Improvement (2005). Management of Labor.

[B23] National Collaborating Centre for Women's and Children's Health (2004). Caesarean Section.

[B24] The AGREE Collaboration (2001). Appraisal of Guidelines for Research & Evaluation (AGREE) Instrument.

[B25] Devellis RF (2003). Scale Development: Theory and Applications.

[B26] Plsek PE (1999). Quality improvement methods in clinical medicine. Pediatrics.

[B27] Smith WR (2000). Evidence for the effectiveness of techniques to change physician behavior. Chest.

[B28] Hayes BE (1997). Measuring Customer Satisfaction: Development and Use of Questionnaires.

[B29] Juni P, Altman DG, Egger M, Egger M, Smith GD, Altman, DG (2001). Assessing the quality of randomised controlled trials. Systematic Reviews in Health Care: Meta-analysis in Context.

[B30] Studnicki J, Remmel R, Campbell R, Werner DC (1997). The impact of legislatively imposed practice guidelines on cesarean section rates: the Florida experience. Am J Med Qual.

[B31] Lagrew DC, Morgan MA (1996). Decreasing the cesarean section rate in a private hospital: success without mandated clinical changes. Am J Obstet Gynecol.

[B32] Batalden P, Al-Assaf AF, Schmele JA (1997). Organizationwide quality improvement in health care. The Textbook of Total Quality in Healthcare.

[B33] Hofmeyr GJ (2005). Evidence-based intrapartum care. Best Pract Res Clin Obstet Gynaecol.

[B34] Allison L (2004). Evidence-based practice as a tool for case management. Case Manager.

[B35] Rhydderch M, Elwyn G, Marshall M, Grol R (2004). Organisational change theory and the use of indicators in general practice. Qual Saf Health Care.

[B36] Turan JM, Bulut A, Nalbant H, Ortayli N, Erbaydar T (2006). Challenges for the adoption of evidence-based maternity care in Turkey. Soc Sci Med.

